# A Dry Membrane Protection Technique to Allow Surface Acoustic Wave Biosensor Measurements of Biological Model Membrane Approaches

**DOI:** 10.3390/s130912392

**Published:** 2013-09-13

**Authors:** Katrin Reder-Christ, Patrick Schmitz, Marian Bota, Ursula Gerber, Hildegard Falkenstein-Paul, Christian Fuss, Marius Enachescu, Gerd Bendas

**Affiliations:** 1 Pharmaceutical Chemistry, Rheinische Friedrich-Wilhelms-Universität Bonn, An der Immenburg 4, Bonn D-53121, Germany; E-Mails: schmitzp@uni-bonn.de (P.S.); ugerber@uni-bonn.de (U.G.); hfalkens@uni-bonn.de (H.F.-P.); cfuss@uni-bonn.de (C.F.); gbendas@uni-bonn.de (G.B.); 2 Center for Surface Science and Nanotechnology, University Politehnica of Bucharest, 313 Splaiul Independentei, Bucharest, RO-060042, Romania; E-Mails: marian.bota@gmail.com (M.B.); marius.enachescu@upb.ro (M.E.); 3 Academy of Romanian Scientists, 54 Splaiul Independentei, Bucharest, RO-050094, Romania

**Keywords:** biosensor, Langmuir-Blodgett technique, membrane protection, SAW, trehalose

## Abstract

Model membrane approaches have attracted much attention in biomedical sciences to investigate and simulate biological processes. The application of model membrane systems for biosensor measurements is partly restricted by the fact that the integrity of membranes critically depends on the maintenance of an aqueous surrounding, while various biosensors require a preconditioning of dry sensors. This is for example true for the well-established surface acoustic wave (SAW) biosensor SAM^®^5 blue. Here, a simple drying procedure of sensor-supported model membranes is introduced using the protective disaccharide trehalose. Highly reproducible model membranes were prepared by the Langmuir-Blodgett technique, transferred to SAW sensors and supplemented with a trehalose solution. Membrane rehydration after dry incorporation into the SAW device becomes immediately evident by phase changes. Reconstituted model membranes maintain their full functionality, as indicated by biotin/avidin binding experiments. Atomic force microscopy confirmed the morphological invariability of dried and rehydrated membranes. Approximating to more physiological recognition phenomena, the site-directed immobilization of the integrin VLA-4 into the reconstituted model membrane and subsequent VCAM-1 ligand binding with nanomolar affinity were illustrated. This simple drying procedure is a novel way to combine the model membrane generation by Langmuir-Blodgett technique with SAW biosensor measurements, which extends the applicability of SAM^®^5 blue in biomedical sciences.

## 1. Introduction

Surface acoustic wave sensors have been developed during the last years as powerful and promising biosensor systems for versatile label-free detection of various biological recognition events, e.g., protein-protein, protein-nucleic acid, or cell-virus interactions [1–6]. This technique is based on piezoelectric properties of quartz sensors. Applying an electrical field to gold coated ST-cut quartz crystal slides, a Love-shear wave is generated at a very thin (5 μm) guiding layer directly deposited at the sensor surface [7]. Consequently, surface binding can be detected by changes of the physical properties of the shear wave. Attachment of components equivalent to a mass increase leads to an angular phase shift [8]. The analytical value is further supported by detecting viscoelastic properties evident by changes in the oscillation amplitude.

Immobilization of one binding partner onto the sensor, as an essential prerequisite can be carried out in different ways. In most cases, proteins and aptamers have been immobilized by carbodiimide chemistry (EDC/NHS coupling) directly onto the sensor, onto a carboxylated dextran layer [9], or indirectly via avidin and biotinylated receptors [10]. Although carbodiimide chemistry offers a simple and reproducible method for protein immobilization, the resulting artificial protein layers can suffer from impeded accessibility of protein molecules and, in general, are relatively far from biological reality, e.g., a biomembrane surrounding.

Biological membranes, composed of a bilayer of mainly phospholipid (PL) molecules, possess not only essential barrier functions to compartmentalize cellular and subcellular components, but are also fundamental elements of biological processes, such as cellular recognition, communication or transport [11]. Consequently, biological membranes are of crucial interest for biomedical sciences.

However, PL bilayers display a highly dynamic and complex system with respect to amphiphilic constituents, embedded protein structures and asymmetrical lateral distribution and organization. In order to focus on certain membrane characteristics for research, the simplification of biomembranes by the use of model membranes has been developed as a vital strategy in biophysical and biomedical research [12–14]. Different techniques have been described to prepare well defined mono- or bilayers of PLs or mixtures of different amphiphiles. In general, based on the amphiphilic structures, the presence of water is an essential driving force for the preparation and integrity of the model membranes.

The most prominent model membrane system in research is still represented by vesicles composed of spherically arranged lipid bilayers [15,16], often used to analyze membrane leakage or transport processes [17,18]. This is a well suited approach in solutions, but vesicles have shortcomings in association with solid analytical supports, e.g., sensor surfaces. Nevertheless, vesicles have been used as membrane models for SAW measurements. A binding of negatively charged vesicles onto the sensor surface, which was positively charged by a poly-L-lysine layer, was shown by Andrä *et al.* for a subsequent peptide interaction with the immobilized vesicles [19]. However, it remained unlikely that the lipid layer was indeed a fused bilayer considering the high amplitude changes.

As an alternative technique, the formation of so called black lipid membranes (BLMs) by a sophisticated preparation procedure is important for the analysis of electrochemical membrane properties as well as transports [20,21]. However, BLMs have certain limitation with respect to their instability and incompatibility with solid surfaces.

**Figure 1.**
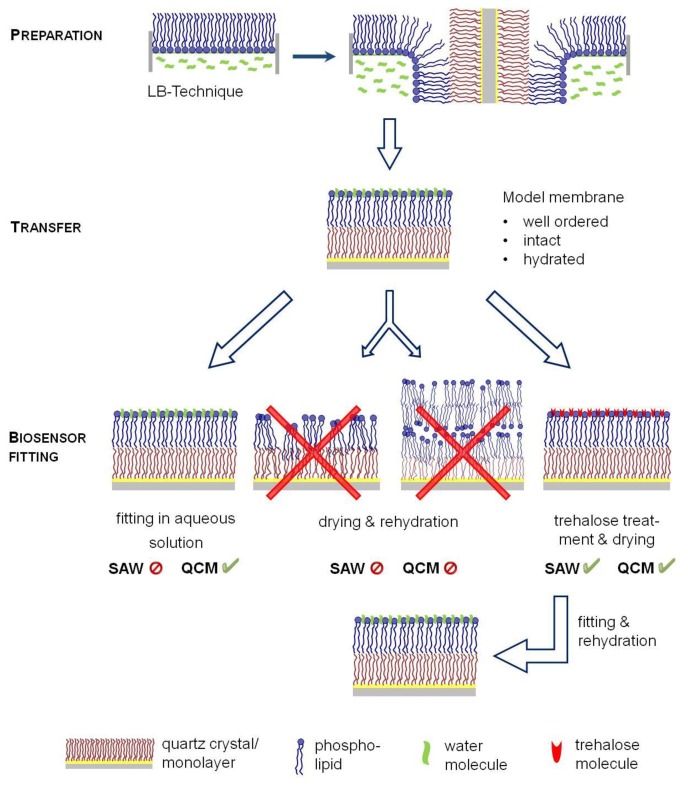
Schematic illustration of model membrane preparation by Langmuir-Blodgett transfer and potential use for sensor measurements. Model membranes can be formed by a monolayer formation at the air/water interface followed by vertically dipping the sensor through this monolayer. The surface attached bilayer requires an aqueous surrounding to maintain its integrity and functionality. Sensor incorporation can be performed either in aqueous solution (QCM) or, as aimed in this study, in a dried state protected by trehalose (SAW and QCM). Drying without protection would lead to non-ordered membranes with functional defects or lipid stacking.

Consequently, biosensor approaches have been combined with another model membrane technology, the Langmuir-Blodgett-technique (LB) (Figure 1), allowing the formation of solid supported membranes [22]. A defined monolayer is formed at a water surface of a Langmuir trough, which can laterally be compressed and transferred to solid surfaces by a defined horizontal dipping of the support material. Consequently, LB technique allows the formation of support-fixed bilayers of variable composition and lateral dynamic properties and is thus the best suited technology to obtain highly defined model membranes. Nevertheless, an important limitation for sensor applications is given by the need of an aqueous surrounding for handling the supported membranes. To avoid a disintegration of the model membrane, an incorporation of the supports into the analytical tool has to be performed under water. This is comparatively simple for a quartz crystal microbalance, but SAW devices, such as the SAM^®^5 blue require an incorporation of dry sensors in the system to circumvent electrical short-circuit and thus antagonized the application of LB technology approaches as yet.

Trehalose, a naturally occurring non-reducing disaccharide is found in high amounts in those organisms that can resist extreme desiccation or cold temperatures [23–25]. This phenomenon is practically applied for freeze drying of proteins and enzymes to conserve structure and functionality by trehalose [26–28]. Furthermore, the activity of trehalose in anhydrobiosis can be extended to protect biological membranes, also referred as “lipo”-protection. Membrane protection by trehalose has primarily been reported for liposomes.

In presence of trehalose lipid layers can undergo de- and rehydration processes without phase transitions and changes in their integrity [29,30]. Lipid vesicles, for example, normally undergo phase separation, fusion and leakage during dehydration and subsequent rehydration. The exact mechanisms of how trehalose can protect the lipid layers remained controversially, so that up to five hypotheses were discussed [31,32]. The sugar molecules can substitute the water by forming hydrogen bonds with polar headgroups of the amphiphilic lipids (water replacement hypothesis, WRH) or they form a scaffold of hydrogen bonds that can bridge the lipid headgroups under dehydration (headgroup-bridging hypothesis, HBH). Trehalose is also discussed to form amorphous glasses that essentially protect lipid layers (vitrification hypothesis, VIH), which has recently been demonstrated as basis to protect support-fixed bilayers too [33]. A concentration of water molecules by disaccharides close to the surface and thus protection against dehydration is further conceivable (water entrapment hypothesis, WEH). According to the hydration forces hypothesis (HFH), sugar molecules are located between opposing membranes, thus reducing the compressive stress in the bilayer maintaining lateral lipid separation. Molecular dynamic studies have shown that most likely a mixture of all these hypotheses contribute to the effectiveness of trehalose.

The present study aims to elucidate whether trehalose could be an option for a simplified drying procedure of sensor-fixed model membranes and the application of the well-defined LB technology for SAW measurements. This would greatly increase the applicability of SAW technology in medical sciences.

## 2. Experimental Section

### 2.1. Chemicals

1-Hexadecanethiol (C_16_H_33_SH) and avidin (from hen egg white) were purchased from Fluka (Neu-Ulm, Germany). 1-Palmitoyl-2-oleoyl-*sn*-glycero-3-phosphocholine (POPC), 1,2-dipalmitoyl-*sn*-glycero-3-phosphocholine (DPPC), 1,2-distearoyl-*sn*-glycero-3-phosphocholine (DSPC), 1,2-dipalmitoyl-*sn*-glycero-3-phosphoethanolamine-N-(biotinyl) (16:0 Biotinyl-PE, sodium salt) and 1,2-dioleoyl-*sn*-glycero-3-[(N-(5-amino-1-carboxypentyl)iminodiacetic acid)succinyl] (18:1 DGS-NTA, nickel salt) were purchased from Avanti Polar Lipids Inc., (Alabaster, AL, USA). D-(+)-Trehalose dihydrate was purchased from VWR BDH Prolabo International (Darmstadt, Germany). Recombinant human VCAM-1 Fc (human IgG1) chimera and human integrin alpha4/beta1 (VLA-4-histag) were obtained from R&D Systems GmbH (Wiesbaden-Nordenstadt, Germany).

### 2.2. Quartz Crystal Cleaning and Biofunctionalization

Cleaning routines of the gold-covered quartz sensors have already been described [34]. According to this protocol, SAW quartz sensors were covered with piranha solution (30% H_2_O_2_:H_2_SO_4_ 1:3) for 2 min, rinsed with ethanol and ultra-pure water (MilliQ™) and dried under an airstream. This procedure was repeated two times. Between the second and third cleaning step, quartz crystals were additionally rinsed with acetone. The cleaned quartz crystals were put into a chloroform solution of 10 mM 1-hexadecanethiol for about 12 h to form a monolayer via self-assembling. The quartz crystals were rinsed again with ethanol and finally dried under airstream.

For biotin/avidin binding all bilayers were prepared with Langmuir–Blodgett (LB) technique. Briefly, a lipid monolayer of POPC ±1 mol·% Biotinyl-PE was spread on the air-water interface of a Langmuir through. After an equilibration period the monolayer was laterally compressed until about 5 mN/m below the collapse pressure were reached. Subsequently, the monolayer covered quartz crystals were vertically driven through the lipid layer forming a bilayer. Bilayer covered quartz crystals were fitted into the measurement chamber of QCM or SAW device as one of follows: (a) The quartz with bilayer was dried at room temperature and inserted into the QCM or SAW chamber; (b) The quartz was kept in aqueous environment, which was replaced by 1.66 μmol/L trehalose solution. After 15 min quartz crystals were taken from the trehalose solution, wept with fresh trehalose solution and stored at 2–8 °C overnight until the trehalose solution was dried. Dried and trehalose protected quartz crystals were fitted either in the QCM or the SAW device; (c) For comparison only in case of QCM: The bilayer covered QCM quartz crystal was completely fitted in aqueous environment into the QCM chamber. For a His-tag-based immobilization of VLA-4 into the membrane, DPPC/20 mol % DGS-NTA was used and similarly transferred to the SAW sensors.

### 2.3. QCM Experimental

QCM measurements were performed using a LiquiLab21 system (ifak e.V., Barleben, Germany). After bilayer preparation and quartz fitting, the measurement chambers were connected to the oscillator and the run was started under temperate conditions (25 °C). The QCM chambers were permanently purged with ultra-pure water (MilliQ™). After reaching a constant frequency, 3 mL of an avidin solution (9 nM) was injected into the flow system via a three-way valve. The system was finally purged with ultra-pure water (MilliQ™) until a constant frequency was reached again. The run was stopped and the binding constants were calculated from the frequency run as described [35].

### 2.4. SAW Experimental

SAW investigations were performed using a SAM^®^5 blue SAW sensor supplied by SAW Instruments GmbH (Bonn, Germany). The POPC/Biotinyl-PE covered quartz crystal was inserted into the tempered flow chamber (22 °C) and continuously rinsed with ultra-pure water (MilliQ™) (flow rate 40 μL·min^−1^). After rehydration, avidin dilution series between 5 × 10^−15^ M and 1 × 10^−6^ M were injected to interact with Biotinyl-PE.

The DPPC/DGS-NTA covered quartz crystal was inserted into the flow chamber and continuously rinsed with PBS containing 1 mM Ca^2+^ and Mg^2+^. After rehydration, 2 μg of VLA-4 His-tag was injected (160 μL). Subsequently, VCAM-1 was injected in dilution series between 1 × 10^−13^ and 1 × 10^−7^ M to interact with VLA-4. All SAW data were determined by the SensMaster^®^ software, the autosampler is controlled by SequenceMaster^®^ (both by SAW Instruments GmbH). Association and dissociation events were detected as phase shifts indicating binding and unbinding events. Corresponding binding kinetics were calculated via non-linear curve fitting using Origin™ (Additive, Friedrichsdorf, Germany) and FitMaster^®^ (SAW Instruments GmbH), (for the underlying mathematics see [36]). The equilibrium dissociation rate constant (*K*_D_) was calculated by *K*_D_ = *k*_Diss_/*k*_Ass_.

### 2.5. AFM Experimental

Atomic Force Microscopy (AFM) measurements have been performed with a home-built AFM connected to a PllPro2 and SPM100 electronics from RHK Technology Inc. As the surfaces were investigated both in dry and wet conditions the scanning technique used was Scanning Polarization Force Microscopy (SPFM) developed by Hu *et al.* [37]. In SPFM an alternating voltage is applied on the conductive AFM tip and the electrostatic interaction between the tip and the sample can be used to maintain the tip at 20 nm from the surface. The tip used was a Pt covered Si AFM tip with a <50 nm tip radius. The AC bias applied was 3 V amplitude at 3 kHz frequency.

### 2.6. Statistics

QCM and VCAM-1 data represent means ± standard deviations of at least three independent experiments. SAW data for avidin binding display one exemplary curve and the corresponding kinetic constants.

## 3. Results and Discussion

### 3.1. The Potential of Trehalose to Protect Model Membranes—First Evidence from QCM Measurements of Biotin/Avidin Recognition

In order to combine biosensor measurements with biological membrane properties, well-ordered model membranes have to be prepared and transferred to the sensor surface. *In situ* techniques, such as immobilization of intact lipid vesicles have different shortcomings leading to less defined membrane structures. Therefore, the Langmuir-Blodgett (LB) technique appears most promising to obtain defined bilayer structures onto the sensors. Since water is an essential driving force for the integrity of model membranes, a permanent aqueous surrounding during membrane preparation and sensor fitting into the device is indispensable to guarantee a hydrated state of the membrane (Figure 1).

LB-membrane transfer can easily be realized for QCM sensors, because they can be fitted into the separate measurement chambers under water. In contrast, SAW sensors have to be dried for incorporation into the device, but membrane dehydration and rehydration starting the SAW run would lead to membrane disintegration with structural defects avoiding exact measurements. Therefore, we aimed to elucidate whether membrane protection by trehalose could be an option for a simplified drying procedure of sensor-fixed LB membranes thus offering a key to apply SAW technology.

To illustrate first the impact of membrane drying damages on sensor measurements and to compare this with classically performed LB technique, we had to apply QCM measurements. The high affinity receptor-ligand system of biotin and avidin was used to test the functionality of the resulting model membranes. POPC model membranes, containing 1 mol % Biotinyl-PE were transferred to the QCM sensors and stored under water for insertion. The dynamic constants of an avidin solution binding to immobilized biotin displayed a strong association rate combined with a comparable slow dissociation rate. This results in an overall binding constant k_D_ in the low nanomolar/high picomolar range (Table 1). Nevertheless, the detected affinity is lower than published data of biotin/avidin interactions, which are in the range of 10^−14^ to 10^−15^ M [38]. This can be explained by the fact that binding partners free and soluble in a reaction compartment, as indicated in the cited studies, display a higher probability and accessibility for binding events compared to the case of immobilizing one binding component at a surface, as indicated here. This will restrict accessibility and thus reduce the binding probability resulting in lower affinity constants.

To illustrate the importance of continuous hydration of the model membranes, identical model membranes were prepared but stored at 2–8 °C until the quartz crystal was dried. After dry incorporation of the quartz, the start of the avidin binding measurement resulted first in a membrane rehydration. However, the data displayed k_D_ values varying in the range between 10^−8^ and 10^−10^ M with very low reproducibility and resulting insentient standard deviations, hence not included in Table 1. This is a clear indication of membrane damages. Thus, the simple drying of the LB films is not suitable for biosensor membrane measurements due to resulting defects in membrane order.

To avoid these membrane defects during de- and rehydration, trehalose was applied as a protectant. Therefore, the aqueous surrounding was replaced by a trehalose solution after LB transfer. Again, quartz crystals were stored at 2–8 °C overnight until the trehalose solution was dried. Protected dried sensors were fitted into the QCM chamber. After rehydrating the membrane by rinsing with water, avidin binding was detected leading to nearly identical binding data comparable to the “originally handled” LB films fitted in aqueous solution as described first (Table 1). This indicates that trehalose is able to protect the membranes. Obviously, the interaction between trehalose and PLs avoid membrane fusion or lipid stacking and helps to maintain the headgroup spacing and thus reduce disintegration and lipid separation [31]. It is questionable whether formation of trehalose glasses is the dominant protectant factor, as described in [33], since we used much lower trehalose concentrations as described by Olivier *et al*. Consequently, our data illustrate that a simple membrane protection by trehalose is an option to protect surface-fixed model membranes in a functional state and thus appear to be promising for its application to SAW technology.

### 3.2. Application of Dried Model Membranes to SAW Technology

**Figure 2.**
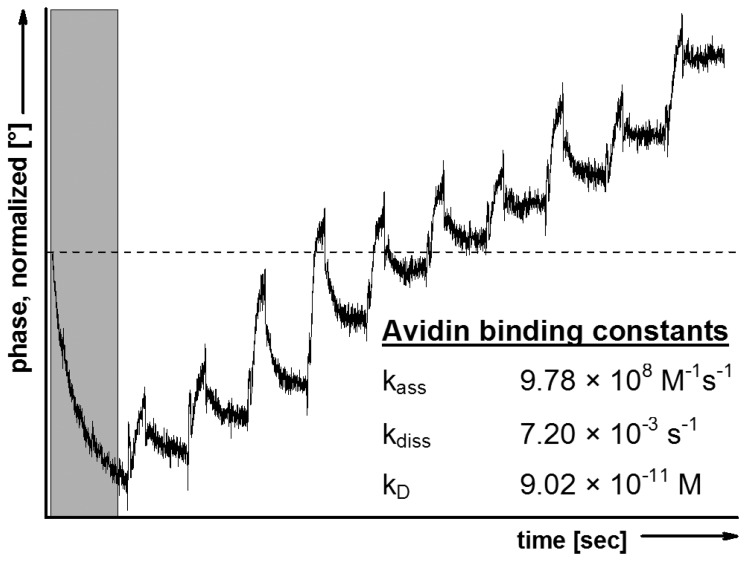
Illustration of avidin binding to POPC/biotinyl-PE covered and trehalose-protected SAW-quartz crystals. Trehalose replacement and rehydration immediately occurs after the run was started (deposited in grey). Avidin injections with increasing concentrations lead to an increase in the phase shifts. Kinetic binding constants, calculated from the SAW-sensorgram, are indicated and display an affinity in the sub-nanomolar range.

To verify the protection procedure outlined above, LB technique und trehalose treatment were transferred to SAW sensors. The trehalose-dried sensors were fitted into the SAW device and the *in situ* rehydration was followed by avidin binding. The kinetic constants of avidin binding to POPC/Biotinyl-PE-membrane, calculated from the sensorgram exemplified in Figure 2, clearly confirm the biological recognition process.

The affinity of binding appears to be somewhat higher compared to that observed with QCM mainly due a much stronger association. These differences originate from the technical circumstances of SAW compared to QCM, *i.e.*, the lower flow rate (40 μL·min^−1^*vs.* 270 μL·min^−1^, respectively) which enables avidin to bind the immobilized biotin at lower shear stress more easily. As a further indication for the specificity of the biological recognition process, an identically dried POPC model membrane, but lacking biotinyl-PE, did not show avidin binding with detectable affinity constants.

The functionality of membrane protection by trehalose was further confirmed by comparing the avidin binding using a biotin-containing model membrane dried in absence of trehalose. The SAW measurement with the resulting phase shifts could not be evaluated with respect to calculate binding affinities. These findings refer to membrane disturbance and strong interference with biological recognition processes.

Altogether, these biological recognition studies confirm that a simple drying procedure can be applied to protect membranes onto a SAW sensor in a dried state and thus allow the combination of defined LB-bilayer preparation with SAW measurements.

### 3.3. Measurement of More Complex Model Membrane Systems by SAW Technique after Trehalose Protection

The biotin/avidin interplay is a high affinity biological recognition system often used for simplification of binding events. In order to evaluate whether the membrane protection procedure is also valid for more complex and physiologically relevant recognition systems, we selected an integrin as a representative of common adhesion receptors in the body interacting with its ligand. The integrin VLA-4 (very late activation antigen-4) is important for leukocyte binding onto the blood vessel wall in term of inflammatory response. Furthermore, VLA-4 binding is also involved in tumor cell metastasis mediating the adhesion of certain cancer cells onto the endothelium by recognizing the ligand vascular cell adhesion molecule-1 (VCAM-1) [39–41].

To date, kinetic constants of VLA-4/VCAM-1 binding were determined applying either VLA-4 and VCAM-1 expressing cells or membrane preparations thereof [4,42]. Although both ways offer the possibility to study the VLA-4/VCAM-1 interaction in their natural membrane surrounding, there are numerous factors that may affect or add to this binding. However, although immobilization of purified molecules on solid surfaces, e.g., by EDC/NHS chemistry could reduce the problem of external effects, a highly complex and heterodimeric structure of an integrin will not easily be immobilized in a functional conformation at an artificial surface without a lipid surrounding.

**Figure 3.**
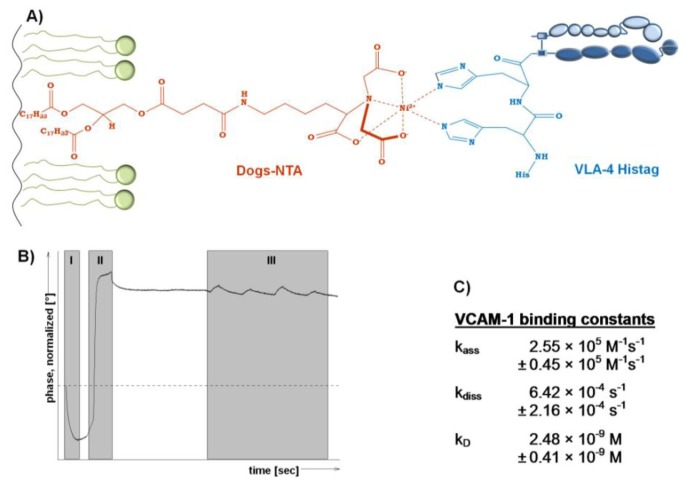
SAW detection of the VLA-4/VCAM-1 binding affinity using a trehalose protected model membrane approach. (**A**) Scheme of an immobilized PL mixture (colored green) with the chelating lipid DGS-NTA to interact with the His-tagged integrin VLA-4 at the membrane surface; (**B**) SAW sensorgram of the reconstitution of the dried membrane and trehalose replacement (I) followed by *in situ* integrin immobilization (II) and interaction with increasing concentrations of VCAM-1 (III) in real time; (**C**) Kinetic calculations from the sensorgram lead to binding affinities in the nanomolar range.

Since the integrin VLA-4 is commercially available as a His-tagged heterodimerized protein, an orientated incorporation into model membranes containing a His-tag chelating lipid appears as interesting option to detect the affinity of binding to the ligand VCAM-1. According to [43], a DGS-NTA containing lipid film was immobilized on the quartz crystal (Figure 3A). However, to use a SAW device for detection, such a model membrane containing the chelator lipid has to be dried protected.

Therefore, a LB-transfer of DPPC/DGS-NTA to a SAW sensor was performed followed by trehalose protection. After insertion of the dried and protected membrane sensor into the SAM^®^5 blue SAW device, the phase changes (Figure 3B) allow to follow all steps in real time. With the start of the measurement the trehalose was replaced and the bilayer was simultaneously rehydrated, which is indicated by the drop in the phase shift in (Figure 3B, I). Next, the His-tagged integrin was injected and tends to bind immediately to the chelating lipid DGS-NTA (Figure 3B, II). Subsequently, VCAM-1 was injected at increasing concentrations to finally detect the binding affinities (Figure 3B, III).

The affinity of VCAM-1 binding to VLA-4 is in the nanomolar range (2.48 nM) and indicates a rapid association combined with a slow dissociation rate (Figure 3C). In general, this finding is in an expected affinity range in comparison to others and thus supports the functionality of the membrane approach. In detail, the VLA-4 immobilization via DGS-NTA within a pure PL surrounding seems to result in slightly higher affinities to VCAM-1 compared to ‘natural occurring’ VLA-4, since the affinity obtained here is fourfold higher than for VCAM-1 binding to immobilized VLA-4 membrane preparations (k_D_ 10.1 nM) [4] and eightfold higher than VCAM-1 binding to VLA-expressing human monoblastoid cell line U937 (k_D_ 80.6 nM) [42]. Both, membrane preparation and intact cells contain various peptides and molecules that can influence the VLA-4/VCAM-1 binding and thus complicate a focused view on the VLA-4/VCAM-1 interaction.

Together, these data indicate that the trehalose protection procedure is easily transferable to more complex model membrane approaches maintaining several biological recognition steps at the membrane, which can be followed in real time by SAW measurements. This greatly increases the spectrum of applications using this device.

### 3.4. Optical Evidence of Trehalose Protection by AFM

The appearance of an intact model membrane after rehydration without any functional defects is a prerequisite for the use of trehalose as protector of LB films. The SAW sensorgrams give advice to an unproblematic rehydration process of LB-films with simultaneous trehalose dissociation and water molecule association (Figure 3B,I). Nevertheless, to find a further confirmation for the protected membrane state by trehalose, we applied SPFM to detect any morphological changes resulting from the membrane drying procedure.

**Figure 4.**
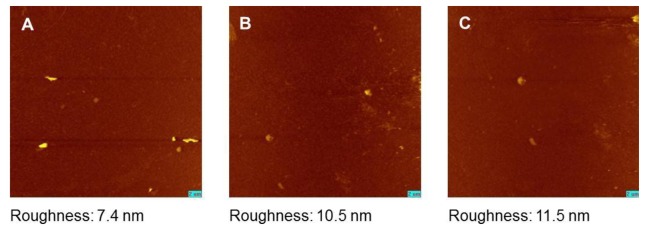
Comparison of trehalose protected and unprotected DSPC model membranes by SPFM. Unprotected DSCP membranes in aqueous surrounding (**A**) are comparable to trehalose protected dry DSPC membranes (**B**) and those after rehydration (**C**). Inserted bars represent 2 μm.

DSPC model membranes were surveyed either in the trehalose treated dry state or after rehydration. Both membranes were compared to DSPC membranes kept in aqueous surrounding without trehalose protection. In all three cases, the membranes appear as even and homogeneous surfaces without any visible defects (Figure 4). This is a further indication that trehalose is suitable to protect the membrane structure without any visible modifications so that rehydration leads to a membrane structure comparable to the non-treated freshly prepared LB bilayer of DSPC. The surface roughness of the membrane as an indicator for any further, non-visible structural defects and inhomogeneities are closely comparable within the preparations.

Nevertheless, we also investigated the non trehalose protected bilayers in a dried and rehydrated state. However, we found no clear indications for membrane disruption and only a marginally increased roughness compared to the data indicated above. This might suggest that the functional constraints of the non-protected bilayers, evidently seen in the sensor measurements, are not caused dominantly by a bilayer fusion, lipid stacking or replacement of bilayer patches from the sensor. One has to consider that the covalently linked first monolayer at the sensor surface obviously stabilize the bilayer structure, which can explain a less susceptibility to structural dehydration damages in comparison to other studies [33].

## 4. Conclusions

Our data indicate that trehalose as a membrane protectant is not only able to maintain the integrity of vesicular membrane structures in a dried state, but also protects surface-supported model membranes during drying. This facilitates the application of technically demanding model membrane preparations for numerous biosensor applications that normally require a dry preconditioning of the sensors. As exemplified here for SAW, the simple protection strategy by trehalose and the resulting intactness and functionality of membranes after rehydration is an important contribution to further establish biosensor measurements, for instance SAW technology, in ambitious biomedical research fields.

## Figures and Tables

**Table 1.** Kinetic constants of avidin binding to POPC/Biotinyl-PE membranes detected by QCM. The preparation of model membranes by LB technology and handling in aqueous solution was compared with a drying step in presence of trehalose. Since the latter resulted in nearly identical affinity data, trehalose appears a promising option to protect surface attached model membranes in a functional state.

	**k_ass_[M^−1^·s^−1^]**	**k_diss_[s^−1^]**	**k_D_[M]**
Aqueous solution	2.36 × 10^5^ ±0.81 × 10^5^	1.00 × 10^−4^ ±0.56 × 10^−4^	4.03 × 10^−10^ ±0.94 × 10^−10^
Trehalose treatment	3.19 × 10^5^ ±0.56 × 10^5^	2.25 × 10^−4^ ±0.37 × 10^−4^	7.24 × 10^−10^ ±1.86 × 10^−10^
